# Evaluation of heterogeneity dose distributions for Stereotactic Radiotherapy (SRT): comparison of commercially available Monte Carlo dose calculation with other algorithms

**DOI:** 10.1186/1748-717X-7-20

**Published:** 2012-02-09

**Authors:** Wataru Takahashi, Hideomi Yamashita, Naoya Saotome, Yoshio Iwai, Akira Sakumi, Akihiro Haga, Keiichi Nakagawa

**Affiliations:** 1 Department of Radiology, University of Tokyo Hospital, Hongo, Bunkyo-ku, Tokyo, Japan; 2 Elekta KK, 3-9-1 Shibaura, Minato-ku, Tokyo 108-0023, Japan

**Keywords:** Monte Carlo, Calculation algorithm, Stereotactic body radiotherapy (SBRT), Lung cancer, Tissue inhomogeneities

## Abstract

**Background:**

The purpose of this study was to compare dose distributions from three different algorithms with the x-ray Voxel Monte Carlo (XVMC) calculations, in actual computed tomography (CT) scans for use in stereotactic radiotherapy (SRT) of small lung cancers.

**Methods:**

Slow CT scan of 20 patients was performed and the internal target volume (ITV) was delineated on Pinnacle^3^. All plans were first calculated with a scatter homogeneous mode (SHM) which is compatible with Clarkson algorithm using Pinnacle^3 ^treatment planning system (TPS). The planned dose was 48 Gy in 4 fractions. In a second step, the CT images, structures and beam data were exported to other treatment planning systems (TPSs). Collapsed cone convolution (CCC) from Pinnacle^3^, superposition (SP) from XiO, and XVMC from Monaco were used for recalculating. The dose distributions and the Dose Volume Histograms (DVHs) were compared with each other.

**Results:**

The phantom test revealed that all algorithms could reproduce the measured data within 1% except for the SHM with inhomogeneous phantom. For the patient study, the SHM greatly overestimated the isocenter (IC) doses and the minimal dose received by 95% of the PTV (PTV95) compared to XVMC. The differences in mean doses were 2.96 Gy (6.17%) for IC and 5.02 Gy (11.18%) for PTV95. The DVH's and dose distributions with CCC and SP were in agreement with those obtained by XVMC. The average differences in IC doses between CCC and XVMC, and SP and XVMC were -1.14% (p = 0.17), and -2.67% (p = 0.0036), respectively.

**Conclusions:**

Our work clearly confirms that the actual practice of relying solely on a Clarkson algorithm may be inappropriate for SRT planning. Meanwhile, CCC and SP were close to XVMC simulations and actual dose distributions obtained in lung SRT.

## Background

Lung cancer is the leading cause of cancer-related death for men in the world [1], and there are no indications of a decrease in the number of these mortalities. Recently, because of the application of computed tomography (CT) for lung cancer screening, the number of patients diagnosed with early-stage non-small cell lung cancer (NSCLC) has increased. At present, stereotactic radiation therapy (SRT) is considered as a therapeutic option in early stage lung cancer for inoperable patients or for patients refusing surgery. SRT has the advantage of being a minimally-invasive procedure, as well as having a relatively short duration of the course of treatment. In addition, SRT continues to show much better outcomes than those of conventional radiotherapy [2,3].

Since lung is the most inhomogeneous site in the human body, it is very important for SRT planning to take into account differences in tissue density in the dose computation and to consider the secondary electron transport accurately. Therefore, the use of heterogeneity correction and different types of algorithms have been reported to significantly influence the accuracy of the absolute dose [4,5].

To confirm safety and efficacy, SRT for lung cancer is still under evaluation in multi-institutional clinical trials. For example, the Japan Clinical Oncology Group (JCOG) conducted a phase II study 0403 of SRT in operable and medically inoperable patients with pathologically proven T1N0M0 non-small cell lung cancer (NSCLC) to evaluate efficacy and safety. Patient accrual for operable cases and their 3-year follow up was completed in February 2010 [6]. Moreover, JCOG 0702, a phase I dose escalation study of SRT in patients medically inoperable or unfit for surgery with pathologically proven T2N0M0 NSCLC, was started to determine the recommended dose. In this context, in JCOG 0403, the prescribed dose was 48 Gy at the isocenter in 4 fractions and heterogeneity corrected doses by pencil beam convolution (PBC) algorithms were used since PBC could commonly be used in almost all clinical practices at that time. However, at the present time it is well known that PBC has shortcomings when it comes to severe inhomogeneities [7,8]. As for lung cancer treatments, the actual dose is to be lower than expected. In JCOG 0702, therefore, the prescription was changed and the planning objective was for 95% of the PTV to be covered by the some isodose (i.e., 50 Gy) with superposition (SP) or other newer algorithm than Clarkson.

Several studies have been conducted on the accuracy of inhomogeneity corrections employing various algorithms. Most of these studies, however, focus on evaluating the dose to the phantom [9,10]. Koelbel *et al*. [11] found that the Pencil Beam (PB) algorithm overestimates the dose to targets in the lung as compared to CCC. These reports were not examined in any real patient. Fragoso *et al*. [12] reported that XVMC is more accurate than PB in SRT. This report, however, compared only two algorithms and was limited for only three patients.

In contrast, the present work summarized four different dose calculations of actual SRT cases with 20 lung cancer patients after dosimetry measurements on an inhomogeneous phantom. The following four dose-calculation algorithms were compared: scatter homogeneous mode (SHM) and CCC available in Pinnacle^3 ^(Philips^©^), SP implemented in XiO (ELEKTA^©^), and XVMC implemented in Monaco (ELEKTA^©^). This study did not include both PB and Clarkson algorithms. Instead, the SHM calculation corresponding Clarkson calculation was employed. Sharing a common plan, dose volume histograms (DVHs) calculated by these algorithms were analyzed to quantify the dose to the targets and lung as an organ at risk (OAR). Furthermore, mean dose, and mean relative difference were employed to assess the algorithms.

## Methods

### Subjects

A retrospective study was conducted on 20 consecutive patients with lung cancer who underwent SRT at the University of Tokyo Hospital from October 2009 to August 2010. The internal target volume (ITV) ranged from 2.3 to 42.2 cc (median, 8.7 cc). Patient characteristics are summarized in Table 1.

**Table 1:** Characteristics of 20 patients

**Case No**.	ITV (cc)	PTV (cc)	Location	
1	9.4	31.4	Rt	S1
2	5.1	22.2	Rt	S6
3	29.8	74.6	Rt	S10
4	8.4	30.3	Rt	S3
5	4.0	17.4	Lt	S1+2
6	9.6	33.0	Rt	hilum
7	16.9	48.5	Lt	S1+2
8	15.6	45.7	Lt	S10
9	8.7	33.6	Rt	S8
10	7.6	29.1	Rt	S8
11	7.5	26.7	Rt	S8
12	41.1	98.3	Rt	hilum
13	9.7	33.3	Rt	S3
14	8.8	30.7	Rt	S8
15	42.2	98.8	Lt	S1+2
16	3.8	16.2	Lt	S1+2
17	2.3	12.2	Lt	S8
18	38.9	85.4	Rt	S10
19	2.3	12.1	Rt	S5
20	6.6	23.0	Rt	S3

Abbreviation; ITV, internal target volume. PTV, planning target volume.

### Treatment planning

CT images for treatment planning were acquired using Aquillion™LB (TOSHIBA^©^) after patients were positioned in a stereotactic body frame (SBF; ELEKTA^©^) in the supine position. An SBF-attached template on the patient's abdomen reduced the mobility of the target [13]. The CT images were acquired with one mm thick slices around the tumor and five-mm-slices elsewhere using the "long-scan-time" technique, which visualized a major part of the trajectory of tumor movement by scanning each slice for a long time [14]. Slow CT scan was performed during four seconds with abdominal compression.

The ITV was delineated on a three-dimensional radiation treatment planning system (3D RTPS) (Pinnacle^3 ^Version 7.4i, Philips^©^) using the lung window of the CT scan for treatment planning. For each case, ITVs and OARs were defined manually slice by slice by the same radiation oncologist. In all cases, planning target volumes (PTVs) were created by adding five mm margins to the ITVs in all directions, and generated using the automatic three-dimensional (3D) contour generation tool of Pinnacle^3^. All calculations were performed with a grid size of 2.0 mm.

Since the JCOG 0403 protocol has involved the older algorithms, such as the PB and Clarkson algorithm, all plans were first calculated with a SHM calculation ignoring a heterogeneous scatter in Pinnacle^3^. The planned dose was 48 Gy in four fractions, using static beams (eight ports); the gantry and couch angles were 180° + 0°, 260° + 0°, 340° + 0°, 30° + 40°, 35° + 320°, 320° + 320°, 30° + 90°, 330° + 90°, respectively. All plans had 6 MV non-coplanar and non-opposing rectangular beams.

In the second step, the CT images and structures, such as PTV and organs at risk (OAR), and beam data were exported to the Xio TPS and the Monaco TPS. Special care was taken to preserve the same exact plan (keeping the monitor units (MUs), beam weights and fixed angles) in all algorithms. Then, three other dose algorithms were used for recalculating: collapsed cone convolution (CCC) from Pinnacle^3 ^ver.7.4i, and superposition from XiO ver.4.4 (ELEKTA^©^), and XVMC from Monaco ver.2.03.01 (ELEKTA^©^).

In Pinnacle CCC dose calculation, to account for inhomogeneity, the kernels are density-scaled during superposition that is performed by using "collapsed cones", which refer to the modeling of a cone in space using a single ray corresponding to the central axis of the cone. The polyenergetic kernels were constructed with Monte Carlo-generated energy deposition cones [15,16]. In the Xio superposition dose calculation method, a fanned grid is created and dose is computed by convolving the total energy released with Monte Carlo-generated energy deposition kernels which are represented in spherical coordinates [17]. For more accurate calculation in inhomogeneous tissues the kernels are allowed to change with the local electron density variations and to reduce calculation time by using the multi-grid superposition method [18]. The XVMC dose engine implemented in Monaco (ELEKTA^©^) was applied in this study. Kawrakow *et al*. [19] developed VMC as a fast calculation engine for electron beams, and VMC was later extended to photon beams as XVMC [20]. The dose calculation cube voxel size and the statistical uncertainty of the XVMC dose calculation in this study were 2 × 2 × 2 mm^3 ^and 1%, respectively. All of the TPS's were installed in the Department of Radiology, University of Tokyo hospital, and were commissioned for a 6 MV photon beam provided by an Synergy linear accelerator (ELEKTA^©^).

### Phantom testing

To validate the reliability of the comparison among various algorithms, it is required to verify the accuracy of beam modeling in each TPS. After a commissioning recommended by vendors, therefore, the verification of each TPS for a small field size was performed on a water phantom (RT-3000-New-Water, R-tech, Japan) and an inhomogeneous phantom with a spherical insert of 3 cm diameter (RT-3000-New-Water with cork, R-tech, Japan), which was placed on the middle of the cork. The isocenter was located on the middle of these phantoms. All calculations were performed with volume for a grid size 2 × 2 × 2 mm^3 ^by using the actual CT images with a CT-to-density table of Aquillion™LB. The comparison was made with a 3 × 3 cm^2 ^single beam in the water phantom and the same eight non-coplanar beams of 4 × 4 cm^2 ^as shown in previous subsection in the inhomogeneous phantom. These results were compared with the measurement at isocenter using a pinpoint ion chamber (PTW, Germany).

### Treatment plan evaluation

The dose distributions were compared with each other. This study also evaluated the shape differences of the dose volume histograms (DVHs) for the different calculations using the same plan. For each treatment plan, the minimal, maximal, and median relative doses in the ITV and PTV were compared and the differences calculated. The paired *t-*test was used to examine the differences in dose volume indices between different algorithms. A probability value of less than 0.05 was considered to be significant.

## Results

### Phantom testing

The result of dose calculation using phantoms is summarized in Table 2. The dose difference from the measurement was less than 1% in all algorithms with a 3 × 3 cm^2 ^field size in the water phantom. The calculation results with non-coplanar eight beams showed that the algorithms except for SHM can reproduce the measurement data very well even though the inhomogeneous phantom was employed. As expected, the SHM algorithm had a large error in the inhomogeneous phantom, where the scattered photons contributed to the dose significantly. The phantom test also revealed no large differences among XVMC, CCC and SP algorithms in the IC dose.

**Table 2:** Comparison of IC doses in four algorithms with the measeured doses

	Pinpoint chamber	SHM	error	CCC	error	SP	error	MC	error
	[cGy]	[cGy]	[%]	[cGy]	[%]	[cGy]	[%]	[cGy]	[%]
Single Beam (3 × 3 cm^2^) in Water Phantom^a)^	124.6	124.0	0.48	124.4	0.16	125.5	-0.72	123.9	0.56
Eight Beams (4 × 4 cm^2^) in inhomogeneous Phantom^b)^	200.9	209.6	-4.33	199.9	0.50	200.6	0.15	201.7	-0.40

*Abbreviations: *SHM, scatter homogeneous mode, CCC, C.C.Convolution, SP, Superposition, MC, Monte Carlo.

a) Single delivery of 200MU from gantry angle of 0 degree.

b) Static 8 beams; the gantry, couch angles and MU were 180° + 0° 33.5MU, 260° + 0° 44.3MU, 340° + 0° 33.6MU, 30° + 40° 34.1MU, 35° + 320° 35.3MU, 320° + 320° 36.1MU, 30° + 90° 34.4MU, 330° + 90° 34.5MU, respectively. The MU employed here has equal weights in IC dose of CCC in inhomogeneous phantom.

### Evaluation of dose distributions in 20 patients

**Figure 1:**
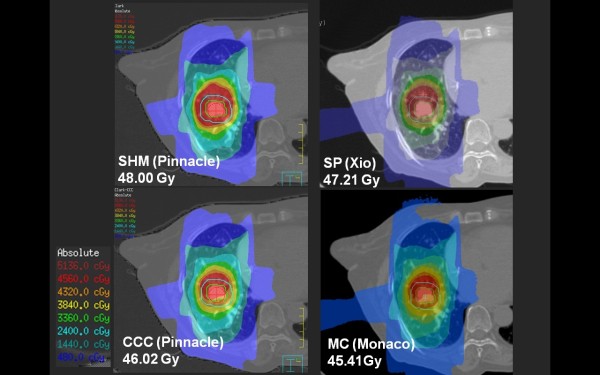
**IC Dose (case 11: Right S8 NSCLC)**. Isodose lines at the isocenter plane calculated with SHM (upper left), CCC (lower left), SP (upper right) and XVMC (lower right) for lung case 11. The ITV (green line) and the PTV (blue line) are shown in the left figure.

**Figure 2:**
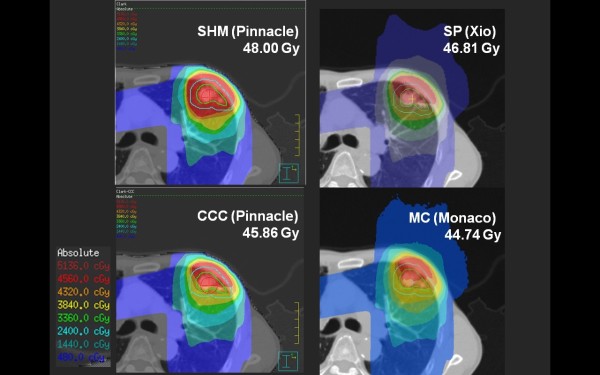
**IC Dose (case13: Left S3 NSCLC)**. Isodose lines at the isocenter plane calculated with SHM (upper left), CCC (lower left), SP (upper right) and XVMC (lower right) for lung case 13. The ITV (green line) and the PTV (blue line) are shown in the left figure.

**Figure 3:**
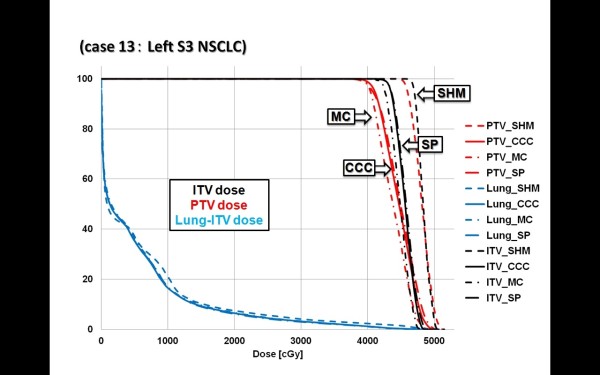
**The cumulative DVHs for the ITV, PTV and (Lung-PTV) using fixed monitor units for case 13**.

Figure 1 shows the dose distributions obtained with SHM, CCC, SP and XVMC at the isocenter plane, with the ITV (green line) and PTV (blue line) in axial view of case 11. Figure 2 also shows the dose distributions in case 13. Although small differences between these algorithms can be seen in these figures, CCC and SP agreed reasonably well in this clinical case (within +/- 2.5% of Dmax) with the XVMC simulations (Table 3). On the other hand, SHM gave overestimated doses in IC and PTV95. This was also observed in the dose volume histograms in case 13 (Figure 3).

**Table 3:** Characteristics and dose-volume parameters of the different dose calculations

Structure	Parameter	SHM	CCC	SP	MC	CCC vs. SP		CCC vs. MC		SP vs. MC	
						
		Mean (SD)	Mean (SD)	Mean (SD)	Mean (SD)	Mean [%]	p-Value	Mean [%]	p-Value	Mean [%]	p-Value
	IC dose(Gy)	48.00 (0.0)	45.35 (1.3)	46.04 (1.4)	44.84 (1.1)	1.49	N.S.	-1.14	N.S.	-2.67	0.0036
	max dose(Gy)	52.17 (2.6)	49.12 (3.1)	48.45 (2.7)	48.10 (2.5)	-1.38	N.S.	-2.12	N.S.	-0.73	N.S.
PTV	PTV95(Gy)	44.92 (0.9)	40.53 (2.4)	41.42 (2.2)	39.68 (2.9)	2.15	N.S.	-2.15	N.S.	-4.39	0.033
	PTVmin	42.88 (1.8)	38.05 (3.0)	38.31 (3.0)	37.57 (2.3)	0.67	N.S.	-1.28	N.S.	-1.96	N.S.
ITV	ITVmin	44.88 (1.4)	41.01 (2.2)	42.38 (2.2)	40.66 (1.8)	3.22	N.S.	-0.86	N.S.	-4.2	0.0094
Lung-ITV	MLD(Gy)	5.72 (2.3)	5.28 (2.1)	5.31 (2.0)	5.33 (2.1)	0.61	N.S.	1.03	N.S.	0.42	N.S.
	V20(%)	9.18 (4.6)	8.22 (4.3)	8.44 (4.3)	8.54 (4.4)	2.55	N.S.	3.68	N.S.	1.16	N.S.
	V5(%)	28.21 (9.5)	27.57 (9.6)	27.85 (9.4)	28.03 (9.6)	1.02	N.S.	1.65	N.S.	0.63	N.S.
	HI	1.21 (0.09)	1.26 (0.09)	1.26 (0.10)	1.27 (0.07)	-0.34	N.S.	0.48	N.S.	0.82	N.S.
	CI	2.69 (0.56)	2.71 (0.38)	2.92 (0.60)	2.86 (0.41)	7.19	N.S.	5.30	N.S.	-2.04	N.S.

N.S., not significant.

*Abbreviations: *SHM, scatter homogeneous mode, CCC, C.C.Convolution, SP, Superposition, MC, Monte Carlo.

PTV, planning target volume. ITV, internal target volume.

HI, homogeneity index. CI, conformity index.

**Figure 4:**
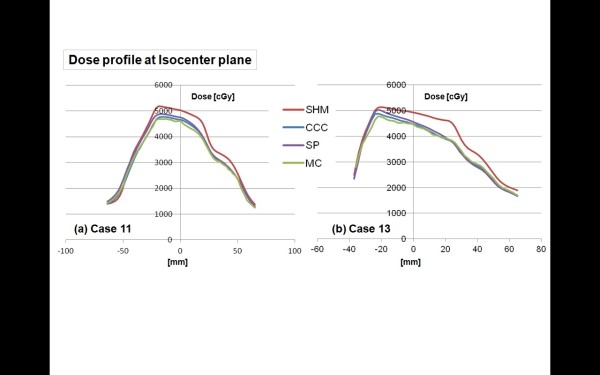
**Dose profiles in the longitudinal (y) direction of case 11 (a) and 13 (b)**. The red, blue, purple, and green curves represent the dose profiles of the SHM, CCC, SP, and MC, respectively.

In order to see the difference among algorithm more clearly, the dose profiles in the longitudinal direction are depicted in Figures 4(a) and 4(b), for case 11 and case 13, respectively. These figures show that all algorithms except SHM estimate the dose to the target in a relatively accurate way, while the SHM gives less good agreement than all other algorithms within ITV. The large discrepancy between SHM and others is mainly caused by the build up and the scatter around the lung/ITV interface. In such a region, the MC algorithm is credible. On the other hand, the fact that the dose profiles of MC and SP are similar each other indicates that the SP algorithm can also reproduce the dose of the lung/ITV interface well.

### Comparison of dose-volume histograms of SHM, CCC, SP and XVMC

The dose distributions for the ITV, PTV, and OAR structures show differences between the four algorithms, and this can be appreciated in the DVH in case 13. Figure 3 shows the DVH comparison of calculations with SHM, CCC, SP and XVMC. The blue lines are for lung dose (Lung-PTV), the black lines for ITV, and the red lines for PTV.

As can be seen in Figure 3, SHM had a tendency to produce higher dose distributions in ITV and PTV than the other algorithms. On the other hand, lung doses (bilateral lung volume minus ITV) were almost the same among all four algorithms. While SHM showed significant differences compared to XVMC in the doses of ITV and PTV, CCC and SP both had similar dose volume histograms to XVMC.

### Differences in dose volume indices

Table 3 lists the mean values and standard deviations of the relative differences in several dose volume indices between the different algorithms. The dose discrepancies between SHM and the others for the median values of the IC dose were -5.5% (median, range: -4.1 to -6.2%) and of the PTV95 were -9.8% (range: -7.8 to -11.7%). Compared to XVMC, SHM greatly overestimated the doses in IC doses and PTV95. The differences in mean doses were 3.16 Gy (6.6%) for IC doses, and 5.24 Gy (10.9%) for PTV95. From this study, an isocentric clinical dose of 48 Gy with Clarkson-type calculation in JCOG 0403 was equivalent to IC 45.4 Gy with CCC, 46.0 Gy with SP and 44.84 Gy with XVMC.

Differences were noted in IC doses between SP and XVMC (-2.67%, *p = *0.0036), and in PTV95 between SP and XVMC (-4.39%, *p = *0.033). We also observed differences in ITV min doses between SP and XVMC (-4.2%, *p = *0.0094). In contrast, we observed no significant differences between CCC and XVMC, and between CCC and SP. No significant differences were found among the four algorithms in conformity index (CI, which is the treated volume, defined as the volume enclosed by the isodose curve of the PTVmin, divided by the PTV volume), homogeneity index (HI, which is the maximal dose divided by the minimal dose), mean lung dose (MLD), V20 (percentage of volume covered by the 20 Gy isodose line) and PTV min.

## Discussion

**Figure 5:**
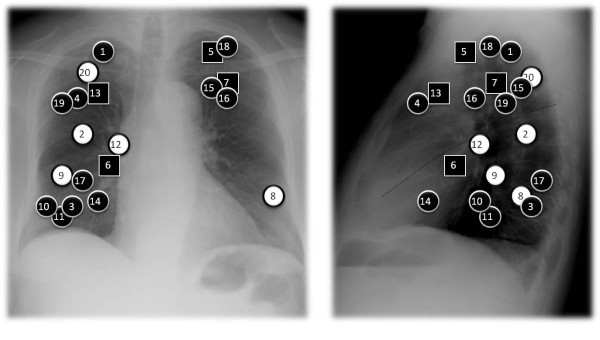
**IC dose differences between SP and XVMC**. Location of each tumor is shown as white circle (dose difference < 2%), black circle (2~4%) and black square (4~5%).

In previous section, the dose distributions of the 20 cases calculated with SHM, CCC, SP and XVMC were compared each other. For a fixed monitor unit normalization, which is considered to be a 'fair' comparison between the algorithms, CCC, SP, and XVMC gave almost the same results on actual patient CT scans. More precisely, no significant differences were observed in lung doses such as MLD and V20. In addition, there were no significant differences between CCC and XVMC, and between CCC and SP. On the other hand, SP gave significantly different results of IC and PTV95 and ITVmin dose from XVMC, that is, SP produced a higher dose than XVMC. This now raises the question of what factors makes these differences. The tumor location and/or ITV volume could be one of the candidates. Although we consider the relationships between these factors and differences, it was not demonstrated clearly to which location and size the differences are significant. For instance, Figure 5 shows that there is a negative association of IC dose between SP and XVMC. On the other hand, the difference of beam modeling for each TPS can be the other candidate to yield the reason why the dose indices in SP are a little bit higher than that in XVMC. As seen in Table 2, comparative measurements have shown that CCC, SP and XVMC were very close to actual measurements in the homogeneous phantom. All agreed with each other within 1.0% accuracy. However, it was also found that the result of SP gave the 1.3% larger dose than that of XVMC in 3 × 3 cm^2 ^square field. From this fact, we concluded that a part of difference between SP and XVMC observed in patient study was caused by the modeling of TPS.

For SRT planning, accuracy in the dose calculation is required because of the heterogeneity in lung tissue. It is acknowledged that CCC can accurately predict the dose distribution [11]. In addition, XVMC methods are potentially the most powerful dose calculation algorithms in radiotherapy [21]. However, due to long calculation times and the huge computational power needed, XVMC algorithms have not seen widespread clinical use [22]. On our TPS, the CCC algorithm (Pinnacle^3 ^ver.7.4i) is approximately 2.5 times faster than XVMC (Monaco ver.2.03) with a small loss in accuracy (10 min vs. 25 min). In addition, we found overall that in SRT planning, the CCC and SP algorithm can reproduce the dose distribution calculated by XVMC very well. Thus, they remain attractive options for routine use in the hospital due to their short computation times.

It is well known that target dose tends to be lower with CCC than with Clarkson. Generally, this implies that pencil beam-like algorithms such as SHM tend to give the wrong impression that a good PTV coverage has been achieved when in reality this is not the case. The reason for this is lateral electron scattering, which is neglected by Clarkson [23]. Therefore, simple algorithms such as SHM especially overestimate the dose in the interface between the target and lung tissue [24]. Our findings have shown agreement with a previous study [9,25]. From this study, an isocentric clinical dose of 48 Gy with the Clarkson algorithm in JCOG 0403 was equivalent to PTV95 dose of 40.5 Gy with CCC in JCOG 0702 (data not shown).

It is concluded that CCC and SP delivered almost the same dose distribution when used in combination open fields like SRT for lung cancer. Our analysis and interpretation is deficient in needing further investigation to ascertain whether CCC and SP are preferable to manage leakage and radiation quality in IMRT.

## Conclusions

This is the first study comparing XVMC with SHM, CCC, and SP for lung SRT treatment plans. The dose distributions in actual CT scans from CCC and SP almost agreed with those of XVMC at energy of 6 MV. An IC dose of 48 Gy with the Clarkson-type algorithm in JCOG 0403 was equivalent to PTV95 dose of 40.5 Gy with CCC in JCOG 0702. We should take careful note of the interpretational problems arising from this discrepancy. As reported in previous studies, our work clearly confirms that the actual practice of relying solely on Clarkson-type algorithm may be inappropriate for SRT planning. The CCC and SP were close to XVMC simulations which we assumed to be the best representation of the real dose distributions in lung SRT. Therefore, CCC and SP are still reliable methods for SRT of lung tumors.

## Competing interests

The authors declare that they have no competing interests.

## Authors' contributions

WT collected and analyzed data and performed statistical analysis. WT and HY drafted the manuscript. NS, YI, AS and AH reviewed the data and revised the manuscript. HY and KN designed the study and revised the final version. All authors read and approved the final manuscript.
